# Supplementary figures and images for: Mimicry can drive convergence in structural and light transmission features of transparent wings in Lepidoptera
Source: eLife. 2021 Dec 21;10:e69080. doi: 10.7554/eLife.69080 (PMC8691843; doi:10.7554/eLife.69080)

# AGNOSIA

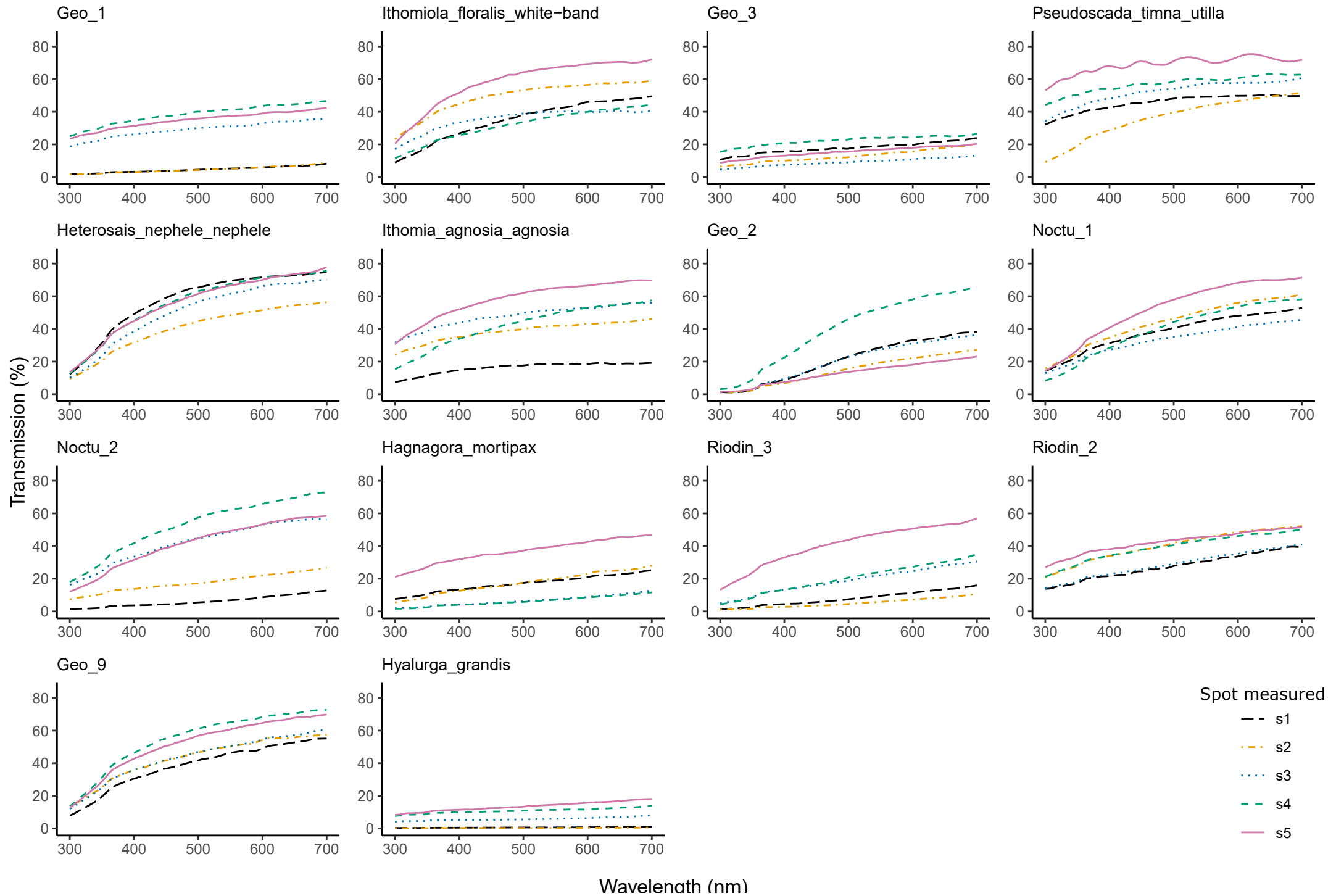

# AURELIANA

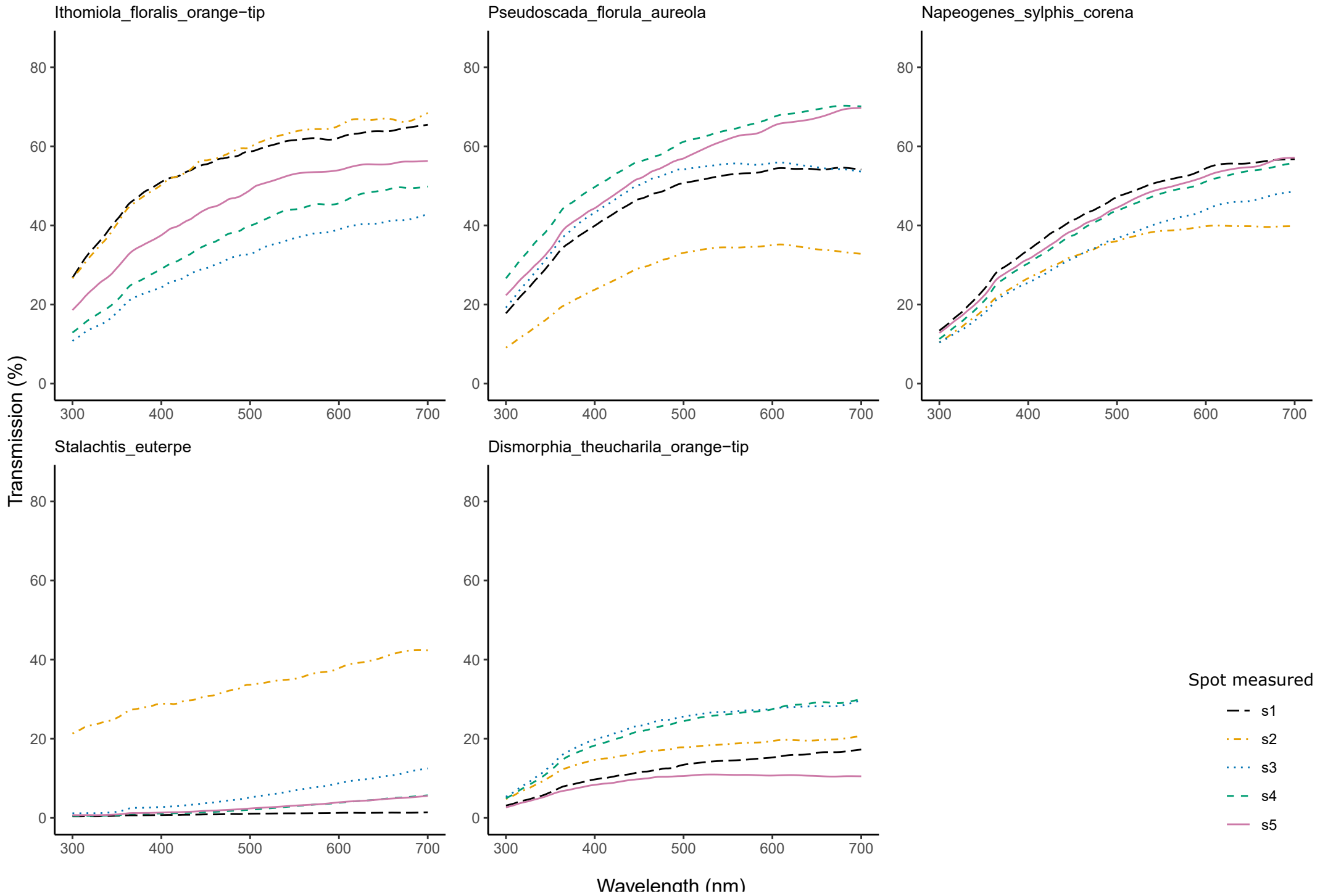

# BANJANA-M

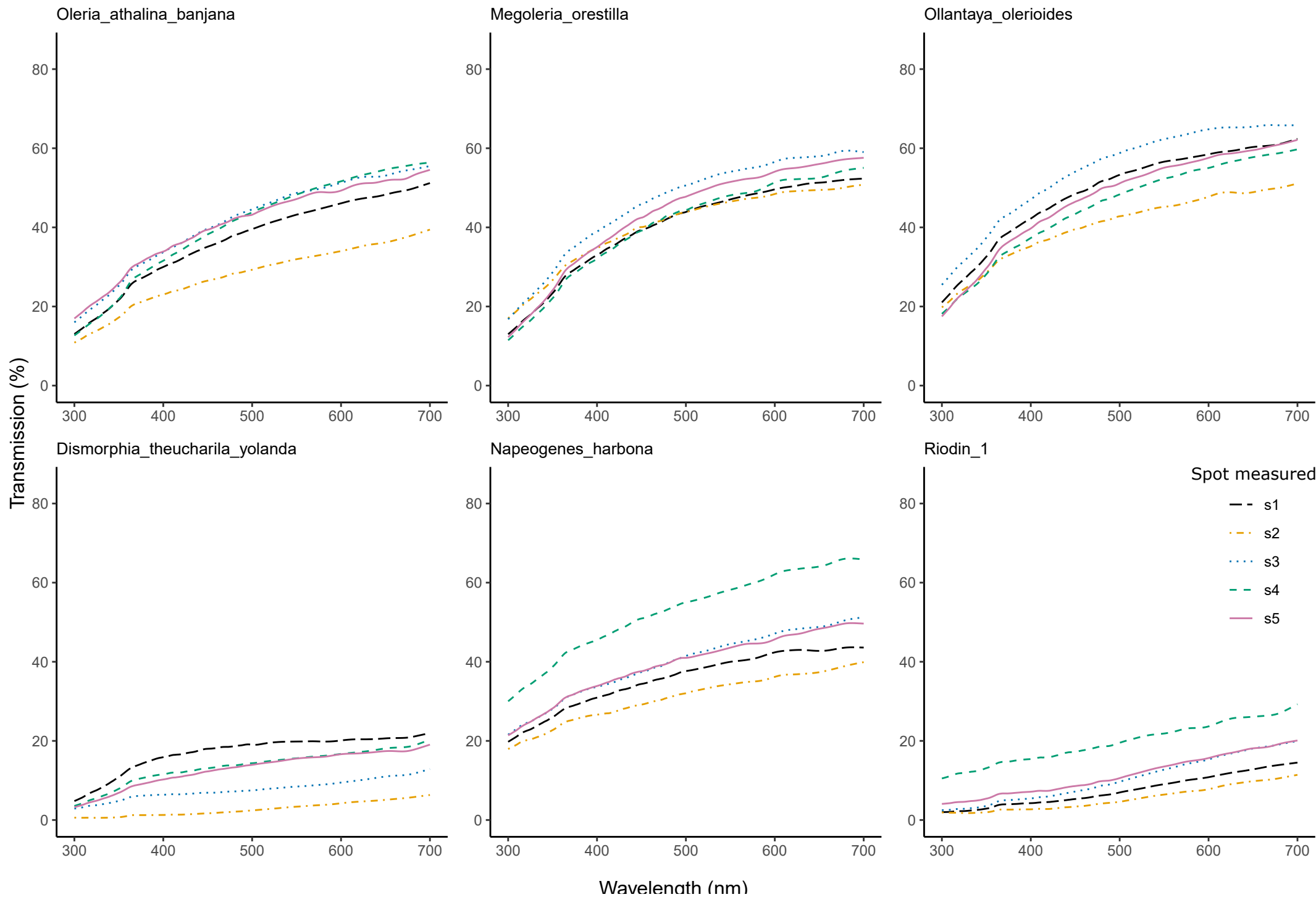

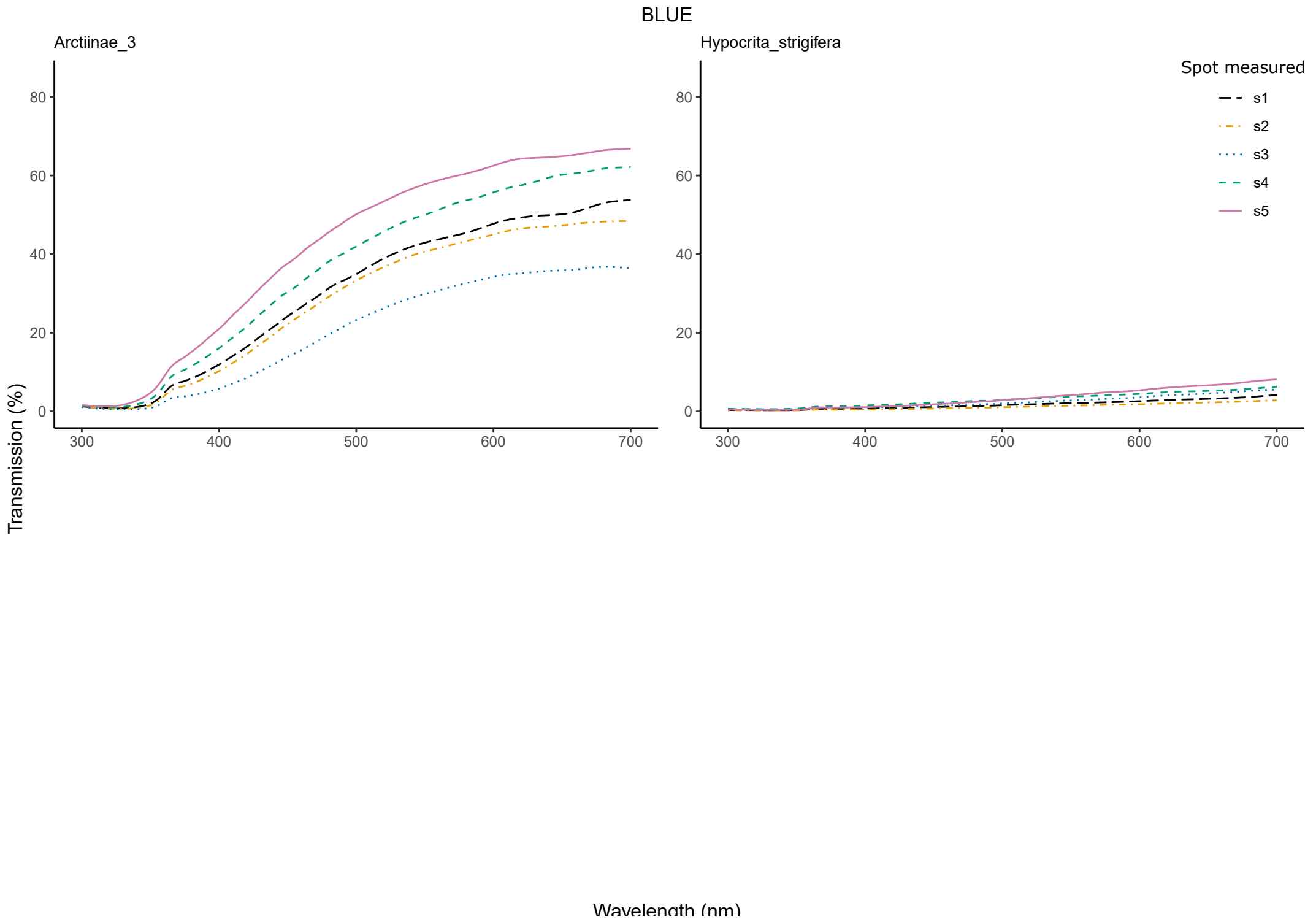

# CONFUSA

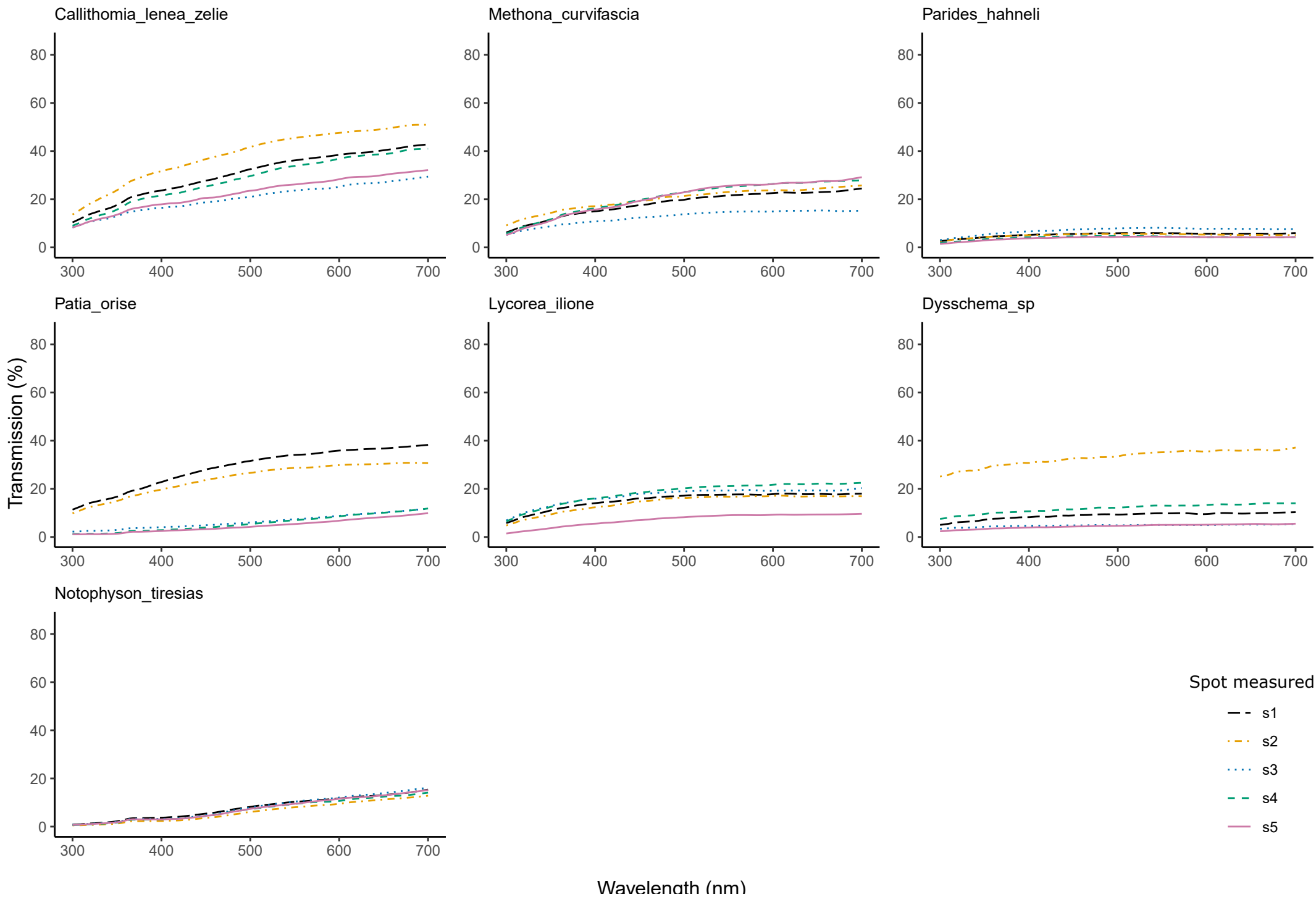

# EURIMEDIA

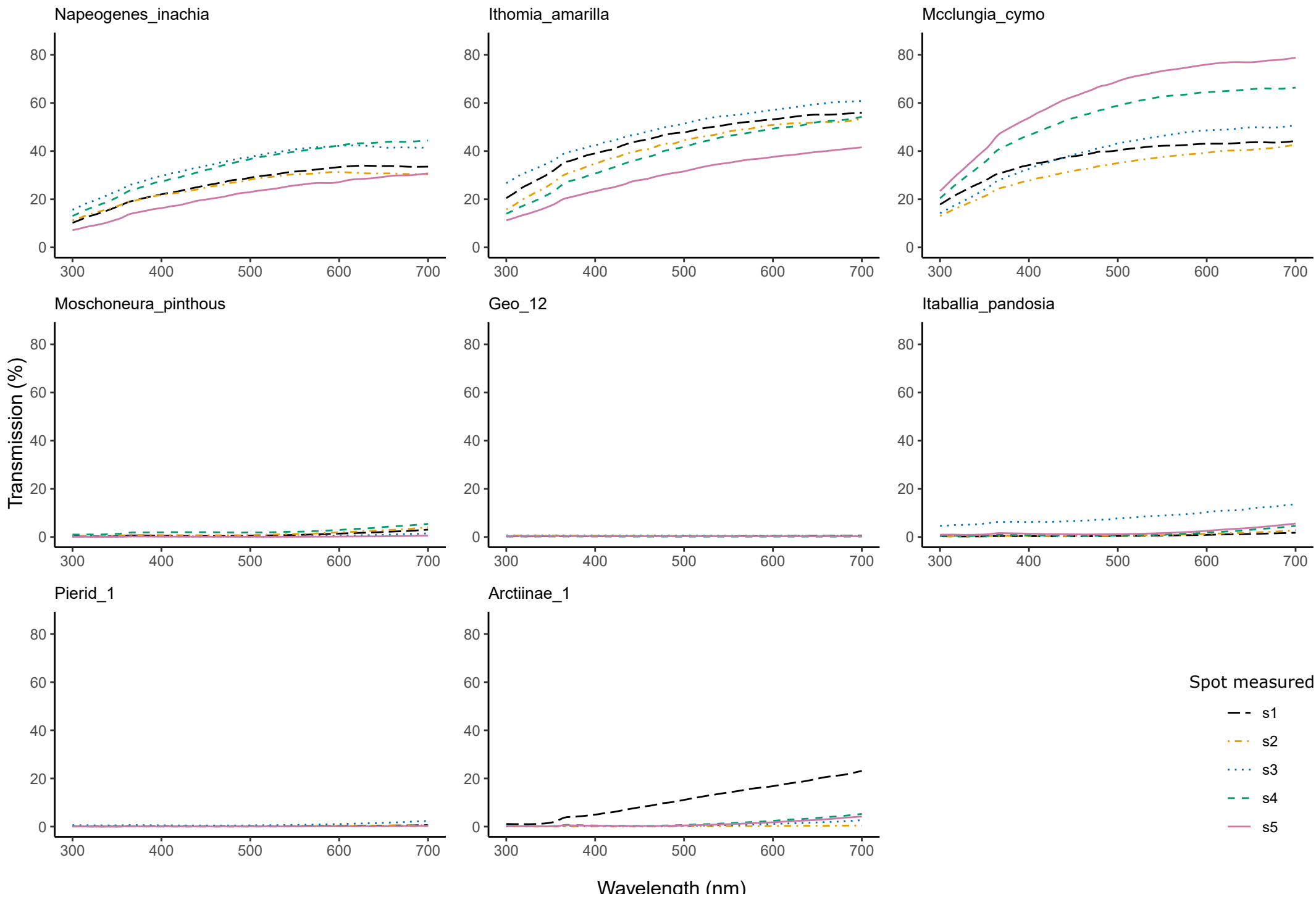

# HEWITSONI

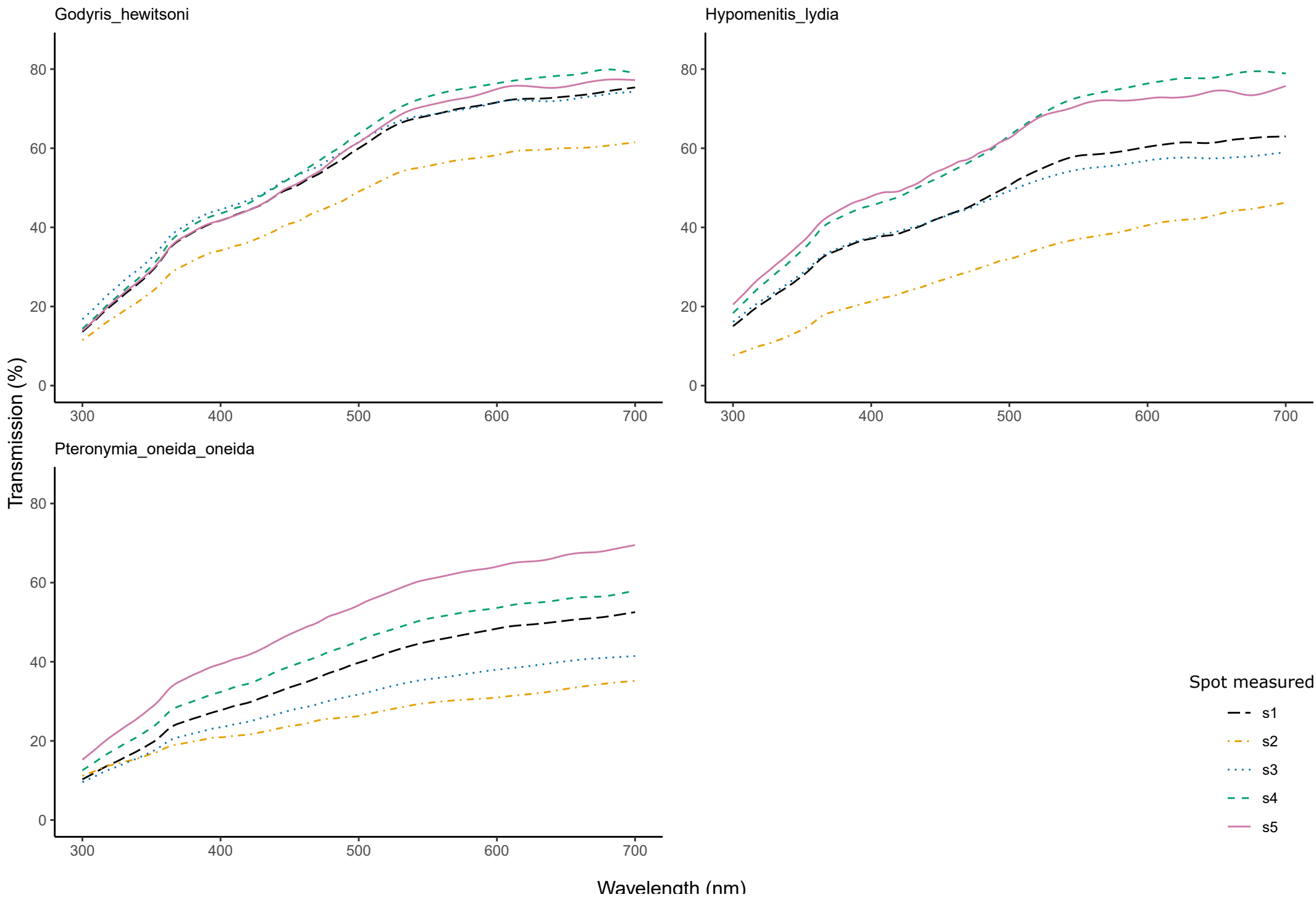

# LERIDA

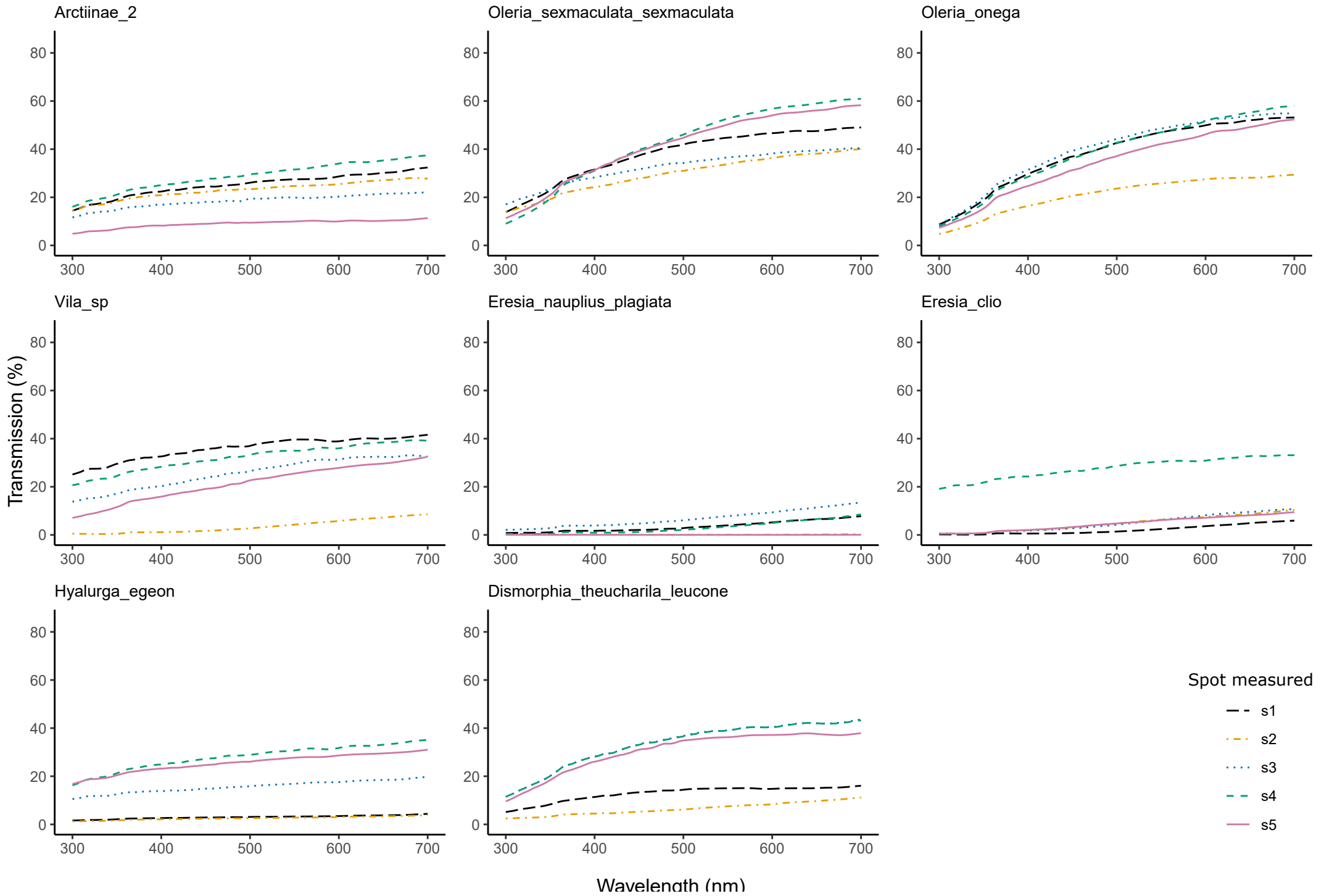

# PANTHYALE

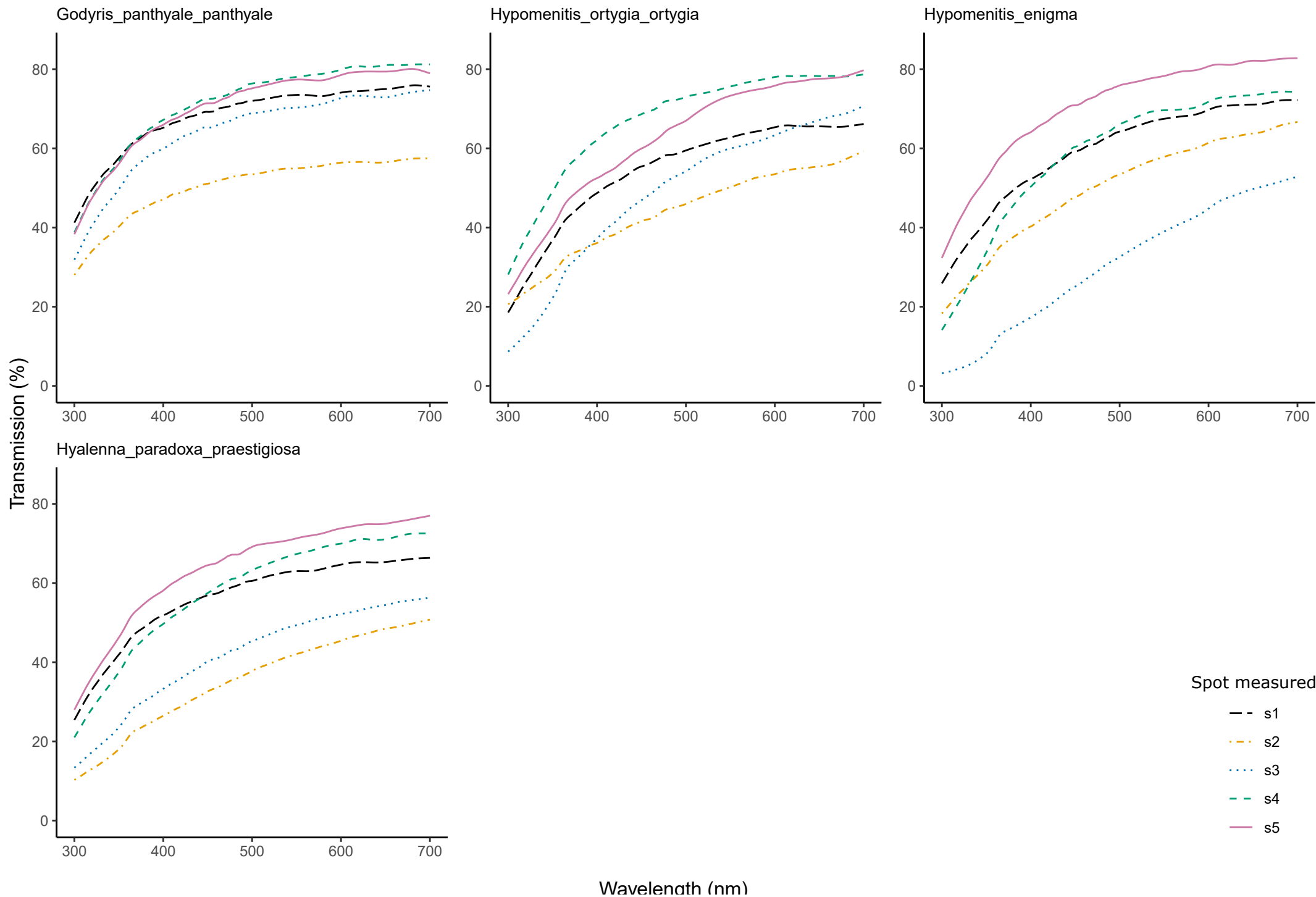

# THEUDELINDA

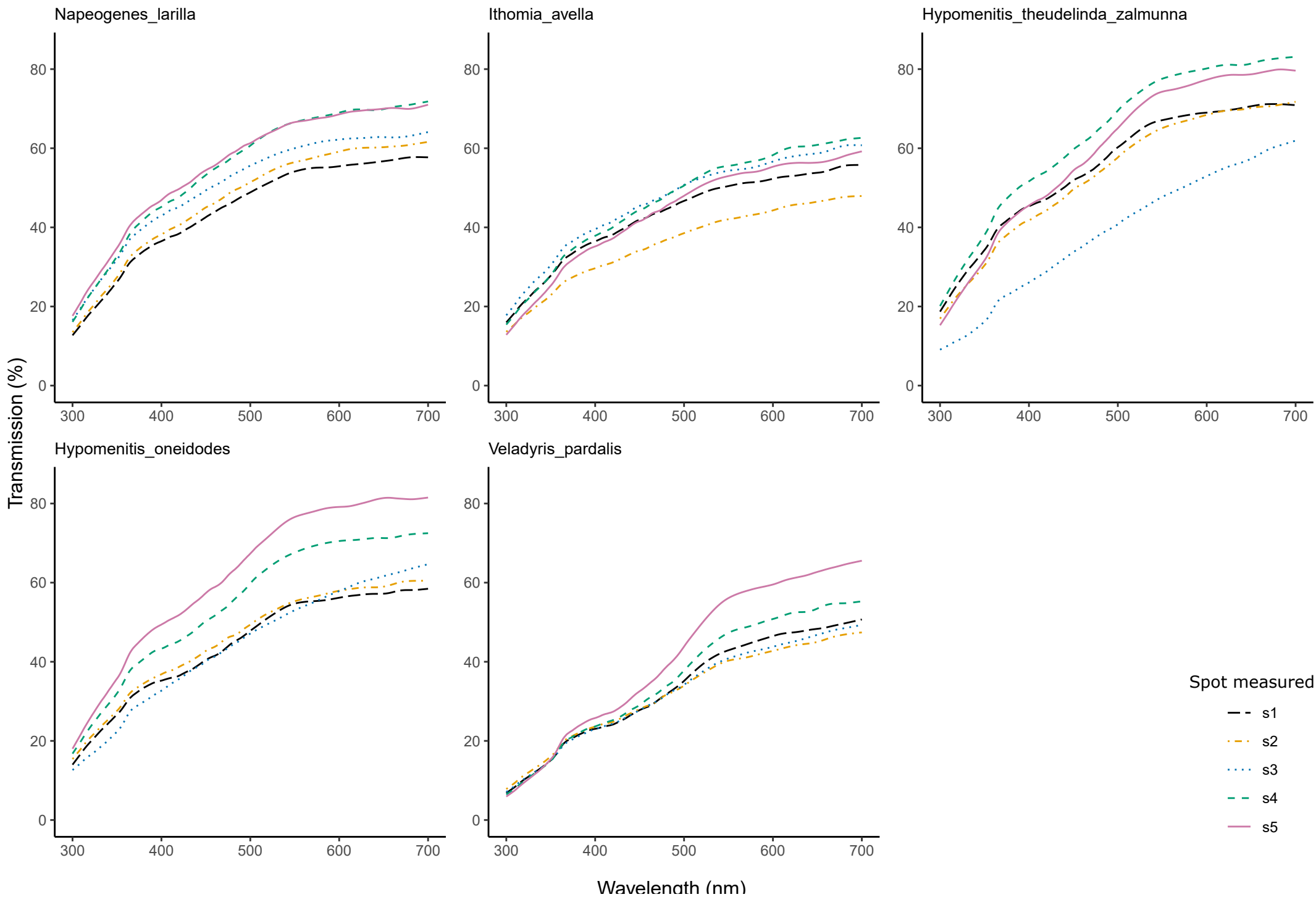

Supplement: Figure 5—source data 1. — For each specimen, the smoothed spectra corresponding to the five spots measured with different colours are shown. Species are grouped by mimicry ring, presented in alphabetical order. The following species are considered as opaque macroscopically: · ‘agnosia’ mimicry ring: Hagnagora mortipax; · ‘aureliana’ mimicry ring: Stalachtis euterpe; · ‘banjana-m’ mimicry ring: Riodin_1; · ‘blue’ mimicry ring: Hypocrita strigifera; · ‘eurimedia’ mimicry ring: Arctiinae1, Geo12, Moschoneura pinthous, Itaballia pandosia, Pieridae1; · ‘lerida’ mimicry ring: Vila emilia, Eresia nauplius plagiata. [file elife-69080-fig5-data1.pdf]
